# Dissecting the Involvement of Ras GTPases in Kidney Fibrosis

**DOI:** 10.3390/genes12060800

**Published:** 2021-05-24

**Authors:** José M. Muñoz-Félix, Carlos Martínez-Salgado

**Affiliations:** 1 Department of Biochemistry and Molecular Biology, University of Salamanca, 37007 Salamanca, Spain; 2 Institute of Biomedical Research of Salamanca (IBSAL), 37007 Salamanca, Spain; 3 Department of Physiology and Pharmacology, University of Salamanca, 37007 Salamanca, Spain

**Keywords:** Ras, kidney fibrosis, fibroblasts, extracellular matrix, MAP kinases, Akt

## Abstract

Many different regulatory mechanisms of renal fibrosis are known to date, and those related to transforming growth factor-β1 (TGF-β1)-induced signaling have been studied in greater depth. However, in recent years, other signaling pathways have been identified, which contribute to the regulation of these pathological processes. Several studies by our team and others have revealed the involvement of small Ras GTPases in the regulation of the cellular processes that occur in renal fibrosis, such as the activation and proliferation of myofibroblasts or the accumulation of extracellular matrix (ECM) proteins. Intracellular signaling mediated by TGF-β1 and Ras GTPases are closely related, and this interaction also occurs during the development of renal fibrosis. In this review, we update the available in vitro and in vivo knowledge on the role of Ras and its main effectors, such as Erk and Akt, in the cellular mechanisms that occur during the regulation of kidney fibrosis (ECM synthesis, accumulation and activation of myofibroblasts, apoptosis and survival of tubular epithelial cells), as well as the therapeutic strategies for targeting the Ras pathway to intervene on the development of renal fibrosis.

## 1. Kidney Fibrosis

Chronic kidney disease (CKD) affects around 10% of the world population. It has high mortality, possibly due to the lack of affordable treatments. CKD may progress to end-stage renal disease, which is treated with dialysis or transplantation [1]. Several cellular processes that contribute to organ faliure take place in CKD: tubular cell death and atrophy, inflammation, and tissue fibrosis [2,3]. Kidney fibrosis is defined as the accumulation of extracellular matrix (ECM) proteins in the renal tissue. Tubulo-interstitial fibrosis involves the accumulation of ECM in the tubular interstitium, between the tubules and the interstitial capillaries [4], while glomerulosclerosis involves the deposition of ECM proteins in the glomerulus, among other cellular mechanisms [5,6].

Depending on the tissue compartment, the source of ECM proteins that are accumulated is different. In the glomerulus, the main ECM-producing cells are parietal epithelial cells, mesangial cells, podocytes and endothelial cells. In the tubular interstitium, the main ECM-producing cells are myofibroblasts and other mesenchymal cells that transdifferentiate into myofibroblasts such as pericytes. Tubular epithelial cells can also produce ECM proteins. In the vessels, vascular smooth muscle cells, endothelial cells and perivascular fibroblasts are ECM-producing cells [7].

Myofibroblasts, referred to in the literature as ‘activated fibroblasts’, are primarily responsible for ECM deposition, with a high ability to generate collagen fibres [8]. Myofibroblasts are rare in healthy kidneys but their abundance increases significantly in chronic and fibrotic diseases. Several cell types are forerunners of myofibroblasts such as resident fibroblasts, pericytes, epithelial cells, endothelial cells or bone-marrow-derived cells [9,10,11].

Apart from the ECM deposition in the glomeruli or in the tubular interstitium, several mechanisms take place during this pathological process such as tubular apoptosis and atrophy, in the case of tubule-interstitial fibrosis, or mesangial cell expansion during glomerulosclerosis [12].

Numerous cytokines are involved in the cellular processes taking place during kidney fibrosis, such as transforming growth factor β1 (TGF-β1), connective tissue growth factor (CTGF) or platelet-derived growth factor (PDGF). TGF-β1 is a pleiotropic cytokine that promotes kidney fibrosis by regulating different mechanisms in different cell types: increases mRNA expression of pro-fibrotic genes in myofibroblasts or tubular epithelial cells, promotes tubular epithelial cell apoptosis [13], regulates immune cell infiltration [14], and promotes epithelial-to-mesenchymal transition [15].

TGF-β1 exerts its cellular functions by activating the canonical-signaling pathway [16], in which TGF-β1 activates its type II receptor (TβRII) which activates the type I receptor (TβRII), known as activin-like receptor 5 (ALK5) and subsequent phosphorylation of Smad2 and Smad3 proteins which in turn activate the profibrotic program [17]. TGF-β1 also activates some non-canonical signaling pathways, with higher relevance in profibrotic gene regulation, such as PI3K/Akt and MAP kinase pathways (ERK, JNK and p38) [18].

## 2. Ras Proteins and Their Role in Cell Signalling

The Ras family of GTPases are small GTP-binding molecules that are products of genes that have been found to be mutated in approximately 30% of all human cancers, and are included in the superfamily of small monomeric GTP binding proteins, acting as molecular switches by cycling between an inactive GDP-bound and an active, GTP-bound state. Because of a defective GTPase activity, the Ras oncoprotein is blocked in the GTP-bound active conformation. In this constitutively active state, Ras induces proliferation, differentiation and oncogenesis [19]. The prototype members of the Ras family are the “classical” Ras proteins (p21 Ras: H-Ras, N-Ras, and K-Ras, highly homologous over the first 85% of their length) together with DIRas, ERas, Gem, MRas, NKIRas, RalA, RalB, Rap, RasD, RasL, Rem, RerG, RRad and RRaS [20]. H-Ras, N-Ras, and K-Ras control and regulate biological functions such as proliferation, differentiation, motility, adhesion, senescence/cell cycle arrest, survival and apoptosis [21]. K-Ras has two alternatively spliced forms derived from Kras-2 gene expression, K-Ras4A and K-Ras4B [22]. A large number of animal and cell biology studies, as well as clinical observations, suggest that these Ras isoforms are not completely redundant in their functions: e.g., in transformed fibroblasts, N-Ras coordinates adhesion, whereas K-Ras regulates motility [23].

**Figure 1:**
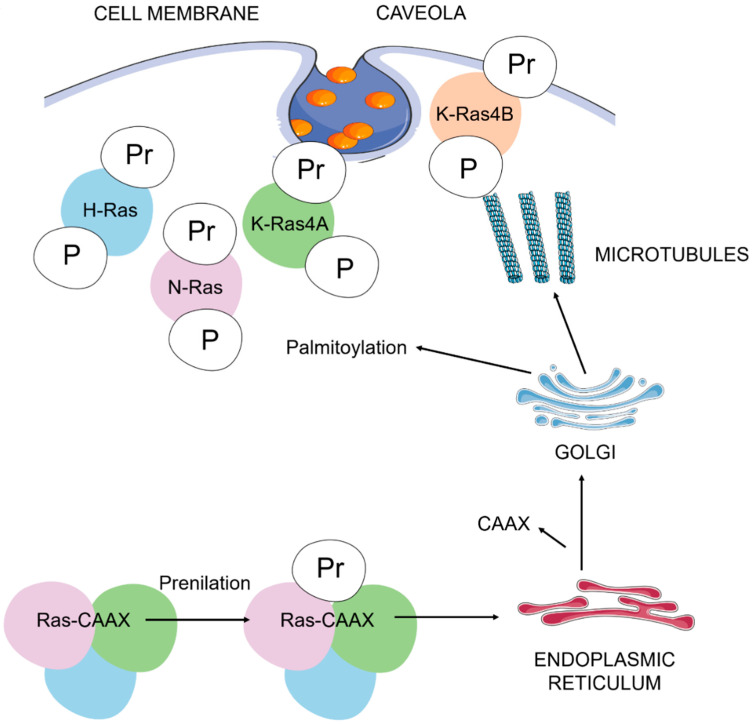
Ras post-translational modifications and trafficking to the plasma membrane. The addition of the carboxyl-terminal prenyl group is mediated by farnesyl or geranylgeranyl transferases. Inside the endoplasmic reticulum, several enzymes catalyze the removal of the AAX residues and the carboxyl methylation of prenylated cysteine residues. Palmitoylation of cysteine residues in H-Ras, N-Ras and K-Ras4A are necessary to complete, via the Golgi complex, their binding to cholesterol-rich plasma membrane pits (lipid rafts, caveola). An unknown pathway completes the binding of K-Ras4B (with a polybasic domain constituted by a lysine-rich sequence) to the plasma membrane. A: aliphatic amino acid; C: cysteine; P: palmitoyl group; Pr: prenyl group; X: serine or methionine. We represent H-Ras in blue, N-Ras in purple, K-Ras4A in green and K-Ras 4B in red.

A wide range of extracellular signals induce Ras activation through binding to protein tyrosine kinase receptors [PTKRs], G protein-coupled receptors (GPCRs) and integrins, among others [22]. Ras proteins also need post-translational modifications: The addition of a C-terminal prenyl group, either farnesyl or geranylgeranyl, is indispensable for the functional anchorage of Ras proteins onto the inner surface of the plasma membrane and for biological activity [24]. K-Ras4B is directly routed to the plasma membrane from the endoplasmic reticulum, but H-, N- and K-Ras4A undergo palmitoylation and traffic by a vesicular mechanism via the Golgi complex. The slow spontaneous GDP/GTP exchange reaction is enhanced by guanine nucleotide exchange factors (GEFs) that promote the formation of the active Ras-GTP state. Intrinsic GTPase activity terminates Ras-GTPase signaling, returning Ras to the inactive GDP-bound state. This GTPase activity is significantly enhanced by interaction with GTPase-activating proteins (GAPs) [25] (see in Figure 1).

In their activated form, Ras proteins stimulate a wide variety of downstream signaling pathways. Raf and phosphatidylinositol 3-kinase (PI3K) were the first two Ras effectors identified and have been the focus of research investigating Ras function. Later, at least 20 effectors have been identified; some of them are GEFs for other GTPases, providing links to different intracellular pathways regulating biological processes such as membrane trafficking, cytoskeletal organization, cell cycle, cell migration and transcription, but the precise number of known Ras effectors is not fully defined [26]. The Raf-1/MAP kinase (MAPK)-ERK pathway promotes cell proliferation and differentiation [27], and it has been considered to be the most important with regard to Ras-mediated transformation of rodent fibroblasts [28]. PI3K pathway activates Akt and thereby promotes cell survival and generates anti-apoptotic signaling [29]. Besides Raf-1 and PI3K, the best-characterized effectors are members of a family of exchange factors for the small GTPase Ral, e.g., RalGDS, which in human cells is directly involved in oncogenesis [28]. Another known effectors are phosphoinositide-specific phospholipase C epsilon (PLCε), T lymphoma invasion and metastasis protein 1 [Tiam 1] and Rac, RAS association domain family (RASSF) [30], RIN1 [31], AF6 (Afadin) [32] and PKCζ [33].

The differences in the function of the different Ras isoforms are also evident in relation to their main effectors: K-Ras is the most powerful Raf-1 activator, followed by N-Ras and H-Ras, and H-Ras is the most potent PI3K activator [34]. K-Ras specifically activates RASSF2 [35] and Rac-1 is differentially activated by H-Ras and K-Ras [36].

Since Ras proteins and their main effectors regulate cell functions, such as proliferation and motility, cytoskeleton and cell adhesion, they have been a topic of interest for fundamental researchers in the field of nephrology, who have considered them interesting candidates to target in pathologies in which kidney fibrosis participates.

## 3. Role of Ras Proteins and Ras-Activated Pathways in the Cellular Mechanisms Taking Place in Kidney Fibrosis

Up-regulated levels of Ras proteins and downstream effectors have been observed in human biopsies and tissues from animal experimental models. These interesting observations evidenced the important role of Ras proteins in kidney fibrosis. Moreover, the high levels of Ras proteins and downstream-activated effectors in different cell types (either in the tubular interstitium or in the glomerulus) have evidenced the involvement of Ras proteins in several processes in each of the different cell types contributing to the development of kidney fibrosis.

Kocher et al. analyzed the expression of the three isoforms (H-, N- and K-Ras) in normal human kidneys and in patients with membranous glomerulonephritis (MGN), IgA nephropathy (IgAN) and IgA-negative mesangioproliferative glomerulonephritis (MPGN). In normal kidneys, they found expression of N-Ras in tubular epithelial cells, K-Ras in mesangial cells, interstitial cells and proximal convoluted tubular cells and H-Ras in all cell types except podocytes. They showed that in the three conditions (MGN, IgAN and MPGN) the three Ras isoforms are present in all cell types except podocytes, being their expression reduced in podocytes [37].

Years later, in the murine model of unilateral ureteral obstruction (UUO), an experimental model of tubule-interstitial fibrosis, Rodriguez-Pena et al. showed an increase in Ras activation during the early response after UUO. Two main pathways activated by Ras, ERK1/2 and Akt, were also upregulated after UUO. By administering U0126, a MEK1/2-ERK1/2 inhibitor and LY294002, an Akt inhibitor, the role of Ras and its effector pathways in the UUO-interstitial fibrosis was functionally demonstrated. Higher levels of phospho-ERK1/2 were detected in the tubular interstitium, associated with myofibroblast proliferation, and higher levels of phospho-Akt were detected in the dilated tubules, associated with cell survival, as a compensatory anti-apoptotic mechanism [38].

**Figure 2:**
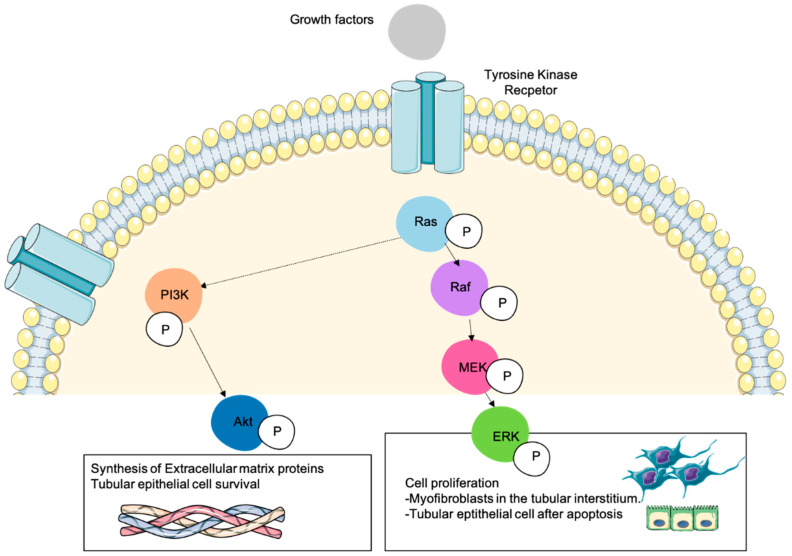
Role of Ras downstream signaling pathways in cellular processes involved in kidney fibrosis. Numerous cellular mechanisms take place during kidney fibrosis by the action of several pro-fibrotic cytokines or growth factors that activate different receptors such as tyrosine kinase. Resident fibroblasts are activated and transdifferentiated into myofibroblasts that proliferate and synthesize ECM proteins. The deposition of ECM proteins and the action of profibrotic cytokines induce renal parenchymal cell death. The activation of Ras can activate the MAPK-ERK1/2 pathway (phosphorylating Raf and MEK), being this the major mechanism that promotes cell proliferation. Resident fibroblasts are transdifferentiated into proliferating myofibroblasts that produce high amounts of ECM proteins. At the same time, several profibrotic cytokines such as TGF-β1 induce tubular cells apoptosis, and some tubular epithelial cells proliferate to counteract this cell death process. All these mechanisms are induced by the ERK1/2 pathway. The regulation (negative or positive depending on the context) of PI3K-Akt by Ras can affect other cellular processes such as ECM proteins synthesis or the promotion of tubular epithelial cells survival. In this figure we represent a Ras-expressing cell during kidney fibrosis in which Ras has been activated by growth factors and its signal has been transduced to their downstream effectors ERK and Akt.

As we indicated above, Raf and PI3K were the first identified Ras effectors. PI3K participates in most Ras signaling pathways [39] and MAPK and PI3K effectors execute cellular signaling programs functioning largely in parallel. Most of the studies analyzing the role of Ras in renal fibrosis have observed that these mediators regulate processes such as ECM synthesis, migration, fibroblast proliferation, etc. (see in Figure 2). There are other intracellular pathways that are also involved in the regulation of Ras-mediated renal fibrosis, as we will see below.

In this section, we will dissect the role of Ras proteins in the processes involved in the development of kidney fibrosis: myofibroblast abundance, especially myofibroblast proliferation—which perhaps is the most relevant mechanism by which ECM producing cells emerge during kidney fibrosis- and epithelial-to-mesenchymal transition (EMT). We will also review the different roles of Ras isoforms in ECM synthesis, a process that directly drives tissue fibrosis.

### 3.1. Effects on Myofibroblast Abundance

There is not a complete and accurate definition of myofibroblasts, but the expression of α-smooth muscle actin (α-SMA) seems to be their best signature. In contrast to normal fibroblasts, they have a large nucleus and electron microscopy detects a rough endoplasmic reticulum [40,41]. These cells have a great ability to generate collagen fibers and are surrounded by ECM proteins [8,42]. Myofibroblasts are the main ECM-producing cells in kidney fibrosis [14].

The origin of myofibroblasts has been controversial for decades. The process of epithelial-to-mesenchymal transition was believed to be an important contributor as a source of myofibroblasts [43]. However, some studies showed that the epithelial contribution to the myofibroblast proliferation was much more limited than expected. LeBleu et al., using the UUO model, demonstrated that the main source of proliferating myofibroblasts in kidney fibrosis emerge from resident interstitial fibroblasts which proliferate (50%). Non-proliferating fibroblasts arise from bone marrow-derived cells [25%]. The endothelial-to-mesenchymal transition program provides approximately 10% of myofibroblasts while the epithelial-to-mesenchymal transition provides only 5% [44]. Moreover, additional mechanisms, such as the partial epithelial-to-mesenchymal transition program were described years later [45].

EMT is a process in which epithelial cells acquire a mesenchymal phenotype and takes place during development and in pathologies such as fibrosis or cancer [46]. During kidney fibrosis, tubular epithelial cells in the renal cortex and corticomedullary region transdifferentiate into mesenchymal cells (activated myofibroblasts) which begin to proliferate [47,48].

Numerous cellular mediators regulate and induce EMT during kidney fibrosis, being TGF-β1 one of the most relevant players. TGF-β1 induces the EMT program in kidney fibrosis through Smad3 activation [49,50]. EMT can be detected during kidney fibrosis by analysing the increase of mesenchymal markers (vimentin, a-SMA) and the loss of epithelial markers such as E-cadherin or ZO-1 [51].

On the other hand, as mentioned above, the proliferation of resident fibroblasts seems to be the main source of myofibroblasts, contributing to the increase in the number of myofibroblasts much more than the epithelial cells via the EMT program. Summarizing, all mesenchymal cells existing in the tubular interstitium are a source of myofibroblasts: resident fibroblasts, pericytes and other mural cells that support the peritubular capillaries as well as vascular smooth muscle cells. In 2008, Picard et al. demonstrated co-localization of α-SMA with the fibroblast marker ecto-5-nucleotidase in the first days after the UUO, demonstrating the transdifferentiation of interstitial fibroblasts to myofibroblasts [9]. Later it was described that in kidney injury, 90% of cells with the mesenchymal marker PDGF-β in co-localization with α-SMA were also positive for myelin protein zero, which is fibroblast-specific, confirming the important contribution of the resident fibroblasts to the myofibroblast population [52]. As mentioned before in the UUO model, more than 50% of the myofibroblasts come from the proliferation of interstitial resident fibroblasts [44]. Thus, fibroblast proliferation seems to be an essential process during kidney fibrosis, and Ras proteins are directly involved in its regulation.

In the following sections, we will describe the role of Ras proteins in the mentioned processes, which cause the emergence of myofibroblasts in kidney fibrosis.

#### 3.1.1. Role of Ras Isoforms in the EMT Program in Kidney Fibrosis

Ras regulates the EMT process induced by TGF-β1, as Grande et al. demonstrated a decrease in UUO-induced kidney fibrosis in mice lacking H-Ras isoform (H-ras^−^), this decrease being associated with a reduction of the EMT program. These authors observed a lower abundance of myofibroblasts in H-ras^−^ mice. In the absence of H-Ras, they detected a higher expression in epithelial markers (E-cadherin) and a reduction in mesenchymal markers (a-SMA and vimentin), demonstrating the involvement of the H-Ras isoform in the EMT process and its consequent regulation of kidney fibrosis [53].

However, although the presence of the EMT process in tubular epithelial cells is easy to demonstrate in vitro, numerous recent studies contradict this in vitro evidence and show that the contribution of epithelial cells in the myofibroblast population during kidney fibrosis is very limited [11,44,54]. The demonstration of the existence of the EMT process in kidney fibrosis in vivo appears to be very challenging, even more, when different mechanisms such as the partial EMT have been described recently [45]. It has been demonstrated that ERK1/2 activation is one of the intracellular pathways responsible for TGF-β-induced EMT [55,56], and seems to be a necessary step in the induction of this process [57,58].

#### 3.1.2. Role of Ras Isoforms in the Renal Interstitial Fibroblast Proliferation

More than 30 years ago, Sharpe et al. described that the K-Ras isoform was predominantly expressed in primary kidney fibroblasts. These authors, using antisense oligos, demonstrated that the K-Ras isoform was playing an important role in cell proliferation, when these renal fibroblasts were stimulated with epidermal growth factor (EGF), fibroblast growth factor, or fetal calf serum [59]. Similar results were confirmed using human renal fibroblasts, showing that K-Ras was the most important regulator of proliferation of the three isoforms. Although H-Ras expression was much lower in these cells, it also played a role in cell proliferation [60].

Years later, Martinez-Salgado et al. observed a lower TGF-β1-induced cell proliferation in mouse embryo fibroblasts (MEFs) lacking N- and H-Ras (N-ras^−^, H-ras^−^) in comparison with their respective controls (N-ras^+/−^, H-ras^+/−^), being this N- and H-Ras induced proliferation a mechanism regulated by the MEK-ERK pathway [61]. Similar results were found in H-Ras deficient MEFs, in which the induction of PCNA or Ki67 expression by TGF-β1 was ablated in H-ras^−^ cells [62]. N-Ras deficiency reduced basal MEF proliferation and TGF-β1-induced proliferation, assessed by cell proliferation assays and analyzing PCNA and Ki67 expression. N-Ras seems to be essential for MAPK-ERK activation and their consequent induced cell proliferation [63]. The absence of K-Ras isoform leads to a strong reduction in fibroblast proliferation associated with lower levels of ERK phosphorylation. K-Ras deficient MEFs showed an increase in phospho-Akt levels, which was associated with compensated cell proliferation [64]. All these results show a very important role of Ras isoforms in the regulation of cell proliferation, being the K-Ras isoform the one with greater weight in this process. The activation of the Ras-downstream MAPK-ERK pathway seems to be the main mechanism involved in the regulation of cell proliferation by H, N and K-Ras isoforms.

#### 3.1.3. Ras Activated Pathways Regulate Cell Proliferation

The involvement of Ras-activated pathways, especially the Raf-ERK1/2 and Akt pathways has been demonstrated in proliferative processes taking place in kidney fibrosis: resident fibroblast proliferation in tubule-interstitial fibrosis, mesangial proliferation in glomerulonephritis and tubular cell proliferation in tubule-interstitial fibrosis. A large number of in vivo studies show the involvement of ERK in the development of fibrosis. We observed Ras-induced ERK activation in an experimental model of UUO-induced tubulointerstitial fibrosis [55], as was shown by other authors [65]. ERK activation seems to be related to tubular proliferation in the obstructed kidney [66]. ERK activation also participates in the proliferative response in rats with experimental glomerulonephritis [67,68]. ERK inhibition suppressed mesangial cell proliferation and reduced the renal damage in a rat model of experimental mesangioproliferative glomerulonephritis [69], and slowed the progression of renal disease in a murine model of polycystic kidney disease [70]. ERK inhibition partially protects cisplatin-induced tubular damage [71], and increased ERK1/2 activation is detected in the kidneys of cadmium-intoxicated rats, probably due to Ras activation [72]. 

There are also numerous in vitro studies evidencing the role of Ras/ERK-mediated intracellular signaling in cell proliferation during the development of fibrosis. ERK1/2 pathway activation is involved in the regulation of TGFβ1-induced proliferation, as we showed that TGF-β1-induced cell proliferation and PCNA expression are reduced in H-ras^−^/N-ras^−^ [61], H-ras^−^ [62] and K-ras^−^ fibroblasts [64] due to the reduced ERK phosphorylation observed in these cells. Elevated ERK1/2 activation may induce cell cycle stop, senescence, apoptosis and differentiation, whereas a slow level of ERK activation is characteristic of proliferative cells [73,74,75,76,77]. Moreover, insulin growth factor-1-induced fibroblast proliferation is also partially regulated by MEK-ERK [78]. ERK activation is also involved in mesangial cell proliferation and transdifferentiation [79]. LDL cholesterol accumulation activates Ras/MAPK intracellular pathway inducing mesangial cell proliferation [80]. ERK is activated by hypoxia/reperfusion in renal epithelial cells, inducing survival and proliferation signaling pathways through the reactive oxygen species-activated EGFR/Ras/Raf cascade [81]. Survival of proximal tubular epithelial cells in mice depends on ERK sustained activation [82]. All these data indicate that ERK is activated in repair processes during ischemia/reperfusion and participates in the control of tubular cell proliferation/apoptosis during the progression of CKD. Moreover, ERK1/2 activation is one of the intracellular pathways responsible for TGF-β-induced EMT [55,56], and seems to be a necessary step in the induction of EMT [57,58].

We also detected Ras activation and Akt phosphorylation in an experimental model of UUO-induced tubulointerstitial fibrosis [38]. Tubular apoptosis actively participates in the deterioration of the obstructed kidney [83] and PI3K/Akt mediates survival mechanisms [84], while Akt upregulation inhibits apoptosis in proximal tubular cells [85]. All these studies confirm the role of Akt activation modulating survival signaling in renal tubules of the obstructed kidney. Akt activation is also increased in a rat model of anti-Thy1 nephritis [86], in diabetic rats [87,88] and in remnant kidneys in rats after 5/6 nephrectomy-induced glomerulosclerosis [89]. Renal ischemia/reperfusion also induces Akt phosphorylation in mice, and the activation of PI3K/Akt signaling maintains cell viability and regulates renal repair after ischemia/reperfusion injury [90]. Besides, erythropoietin treatment decreases renal damage by preventing epithelial cell apoptosis, and this antiapoptotic effect is dependent on Akt phosphorylation by PI3K [91]. PI3K/Akt pathway is also activated by ischemia/reperfusion in the kidney [81,92,93,94,95], thus inducing survival and proliferation of tubular epithelial cells [81].

### 3.2. Regulation of ECM Protein Synthesis

#### 3.2.1. Ras Proteins as Regulators of ECM Protein Synthesis

The common histological correlate and end-point of CKD is renal fibrosis, which is defined as an excessive, pathological accumulation of ECM. Several changes occur in the ECM composition in glomerulosclerosis and tubulointerstitial fibrosis. In glomerulosclerosis, there is an increase in the components of the mesangial matrix (col IV, col V, fibronectin and laminin), the glomerular membrane becomes thicker and an increase in Bowman’s capsule takes place. On the other hand, in tubulointerstitial fibrosis, the tubular basement membrane becomes thicker, and a massive increase in interstitial fibrosis takes place (with increases in collagen I, II, V, VI, VII, XV, fibronectin, biglycan, decorin and versican) [96,97].

In vitro studies using knockdown of Ras isoforms have demonstrated an interesting role of Ras isoforms exerting a negative regulation on the synthesis of ECM proteins. Martinez-Salgado et al. demonstrated that the absence of N- and H-Ras isoforms lead to increased collagen I and fibronectin synthesis [61]. Further studies dissecting the different isoforms confirm that ECM protein synthesis is negatively regulated by H-Ras [62], N-Ras [63] and K-Ras [64].

#### 3.2.2. Ras Activated Pathways Regulate ECM Deposition

Raf, MEK, ERK1, ERK2 and the ERK effector Rsk are expressed in all nephron segments [98]. Downregulation of ECM gene expression is a common target of oncogenic signaling through the Ras/Raf/ERK pathway in 3T3 fibroblasts; repressed genes after Ras-fibroblast transformation includes genes involved in ECM synthesis: fibronectin, several collagen isoforms, lysil oxidase, etc., suggesting that the repression program elicited by Ras and Raf is overlapped with the TGF-β-induced panel of transcripts [99].

Numerous in vitro studies have demonstrated the relationship of the Raf/ERK pathway with the synthesis of ECM proteins. Raf/ERK pathway regulates TGF-β-mediated ECM synthesis, with different effects depending on the cell types and conditions [100,101,102,103,104]. ERK 1/2 inhibits collagen type I synthesis in human fibroblasts and mesangial cells [105]. The ERK pathway is involved in TGF-β1-mediated collagen type I transcription [103]. Our group has shown that the absence of H-, N-Ras [61] and K-Ras [64] isoforms reduce ERK activation and increases fibronectin and collagen expression in fibroblasts. ERK activation is related with an excess of fibronectin synthesis [77], and Ras induction of superoxide activates ERK-dependent fibronectin expression in rat mesangial cells [106]. The activation of the Ras/MEK/ERK pathway is necessary for the TGF-β2-induced activation of CTGF, and the antifibrotic effects of prostacyclin derivatives are due to MEK/ERK inhibition; therefore, the specific MEK/ERK inhibition in fibroblasts prevents fibrosis without interfering with other physiological effects of TGF-β [107]. Ras/MEK/ERK seems to be involved in CTGF gene regulation in mesangial cells [108]. TGF-β1-induced CTGF expression, activity and secretion were reduced by inhibiting Ras and MEK activation in human proximal tubular epithelial cells [109], thus suggesting that inhibiting Ras/MEK/ERK signaling pathways could be a therapeutic strategy in renal fibrosis. ERK 1/2 downregulates MMP-2 activity, as the inhibition of ERK phosphorylation fosters pro-MMP2 and membrane type 1-MMP activation, and reduces tissue inhibitor of metalloproteinase-2 synthesis in mesangial cells [110]. The accumulation of LDL cholesterol within glomerular mesangial cells activates the Ras–MAPK signaling cascade leading to matrix deposition [80]. Repeated tubular ischemia is a usual process in chronic renal fibrosis [111], and renal ischemia induces ERK activation [112,113], being this activation a modulator of renal ischemia/reperfusion injury [92,114].

The role of the PI3K/Akt pathway in the regulation of fibrotic processes has been documented in numerous in vitro and in vivo studies. PI3K/Akt activation is related to increases in ECM synthesis, as PI3K inhibition decreases collagen type 1 and fibronectin expression [99]. Ras-PI3K inhibition also attenuates diabetic nephropathy reducing the accumulation of fibrosis-related proteins [115]. Our group observed that the increases in ECM accumulation were detected in the absence of Sos1 [116], H-Ras [62], N-Ras [63], H and N-Ras [61] and K-Ras [64] in KO fibroblasts is due to Akt activation. Akt activation regulates the increases in TGF-β-induced collagen mRNA synthesis in the lung [117] and in the PDGF-induced collagen synthesis in cultured fibroblasts [118].

All these studies evidence a multifunctional role of Ras proteins in the different processes involved in kidney fibrosis. Several of these effects are cell type-dependent and have been validated only in in vitro studies or in vivo descriptive experiments.

## 4. Functional Validation of the Role of Ras Isoforms in Kidney Fibrosis Development Using In Vivo Studies

With all this evidence showing the involvement of Ras-GTPases in the regulation of kidney fibrosis, several studies functionally validated these Ras-GTPase-induced effects. Some studies were performed in animal models targeting Ras proteins using genetic ablation, anti-sense oligonucleotides, pharmacologic inhibition of Ras effectors, etc., showing the overall effects of Ras proteins in kidney fibrosis development and some interesting potential targets for improving kidney fibrosis therapies.

In 2009, one study demonstrated that mice lacking N- or H-Ras isoforms (N-ras^−^ and H-ras^−^) did not show any differences in the early changes associated to the UUO model: tubular dilatation, accumulation of myofibroblasts, extracellular matrix deposition and apoptosis. These findings showed that although the activation of pan-Ras and the downstream signaling pathways ERK1/2 and Akt were activated in the tubular interstitium and dilated tubules respectively, neither N- and H-Ras isoforms were involved in the early changes induced by UUO and suggested the putative role of K-Ras in these changes and further kidney fibrosis [119]. However, when the UUO was maintained for 15 days, H-ras^−^ mice develop lower kidney fibrosis, associated with a lower abundance in myofibroblasts (α-SMA and vimentin-positive) due to an impaired EMT process in absence of H-Ras [53]. The role and expression levels and role of K-Ras isoform in kidney fibrosis were first studied in the UUO model in rats. In vivo K-Ras knockout models are not viable because the K-ras gene is essential for embryonic development [120]. K-Ras was knocked down using antisense oligonucleotides (ASO), resulting in a decrease in kidney fibrosis associated with a decrease in α-SMA+ myofibroblasts. UUO increased K-Ras mRNA levels. However, the levels of fibroblast specific protein (FSP1) positive cells were upregulated after K-Ras knockdown, suggesting the expression of this marker in inflammatory cells such as macrophages [121]. To delve into the role of the K-Ras isoform, they performed an experimental CKD model by administration of folic acid in mice. They demonstrated that the ASO-induced knockdown of K-Ras isoform led to a 50% reduction of folic acid-induced kidney fibrosis, assessed by Masson’s trichrome and Sirius red. K-Ras knockdown also prevented renal failure evaluated by plasma creatinine levels and BUN [122]. In vivo experimental models of kidney fibrosis have demonstrated a clear involvement of H-Ras, and perhaps a more relevant role of K-Ras in kidney fibrosis [53,121]. The absence or inhibition of these isoforms leads to reduced kidney fibrosis, which is always associated with a reduced number of myofibroblasts, suggesting that the main role of these Ras isoforms in kidney fibrosis is the induction of proliferation of local resident fibroblasts or activated myofibroblasts in the tubular interstitium.

**Figure 3:**
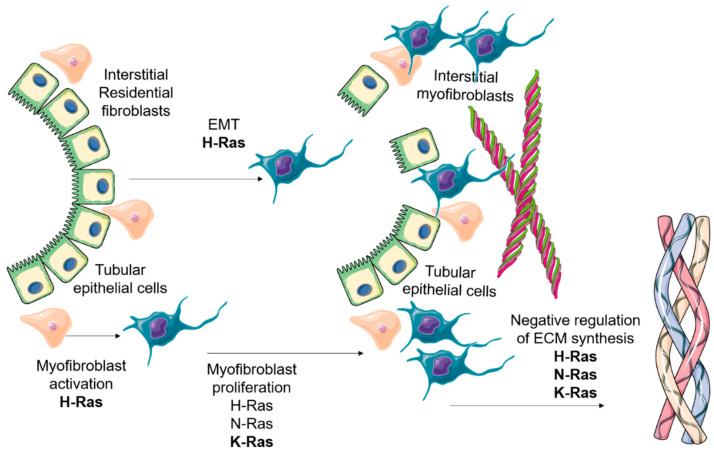
The overall role of Ras isoforms in kidney fibrosis development. As stated above, the main role of Ras isoforms in kidney fibrosis is the promotion of an increase in myofibroblast abundance in the tubular interstitium. The main source of myofibroblasts in this pathological scenario is the local resident fibroblasts and other mesenchymal cells. H, N and particularly K-Ras are involved in myofibroblast proliferation. Several studies have shown the role of H-Ras in the activation of myofibroblasts, due to its regulation of the EMT program. Once expressed in activated myofibroblasts, H, N and K-Ras foster a negative regulation on ECM proteins synthesis. On the left side, we show tubular epithelial cells which undergo EMT after the profibrotic stimuli. At the same time, myofibroblasts are activated in the tubular interstitium and proliferate to synthesize high amounts of ECM proteins, which are accumulated in the tubular interstitium (represented on the right side).

All these studies have corroborated the role of Ras isoforms in experimental models of fibrosis. Although more studies and evidence on the role of the N-Ras isoform are needed, H-Ras and K-Ras seem to have a clear effect on kidney fibrosis development. Both isoforms are involved in the emergence of myofibroblasts in the tubular interstitium: H-Ras regulating EMT process and promoting myofibroblast proliferation, and K-Ras regulating myofibroblast proliferation. The dissection of the cellular processes in in vitro studies have elucidated the interesting role of these isoforms in the regulation of ECM proteins (see Figure 3). Moreover, future studies in glomerular pathologies, which show renal fibrosis, will be very interesting for a better understanding of Ras proteins in kidney disease.

Despite the progress in the understanding of the role of Ras proteins and Ras effectors in kidney fibrosis development, there are several missing links that need to be further studied, especially the participation of mechanisms that have been recently known to be major contributors to kidney fibrosis: the putative role of Ras in other mesenchymal cells such as pericytes or vascular smooth muscle cells, its possible role in the emergence of fibroblasts via other mechanisms such as the endothelial-to-mesenchymal transition, the transdifferentiation of myofibroblasts from macrophages or bone marrow-derived cells or additional regulatory mechanisms of kidney fibrosis based on epigenetics or microRNAs.

Besides, the knowledge of the mechanisms by which Ras regulate kidney fibrosis will be useful to understand other fibrotic pathologies in the liver, lungs, skin or even in the tumor microenvironment.

## 5. Potential Therapeutic Approaches Targeting the Ras Pathway

### 5.1. Antisense Oligodeoxynucleotides (ASO)

ASO are small single strands of approximately 20 oligodeoxynucleotides, which are complementary to the target mRNA sequence. Antisense technology has become a powerful tool used in basic research and promising in clinical therapy [123]. As we have mentioned previously, K-Ras isoform has been knocked down using ASO in in vivo experimental models, showing a strong reduction of kidney fibrosis in rats after the UUO model [121] or mice after folic acid administration [122].

### 5.2. Inhibition of the Ras Pathway by Inhibiting ‘Upstream’ Activation by Angiotensin II

The administration of angiotensin II was found to induce the activation of the small GTPase Ras and their downstream pathways ERK1/2 and Akt in the UUO model. The inhibition of angiotensin II receptors using losartan was found to reduce the synthesis of ECM proteins (fibronectin) and the presence of myofibroblasts (assessed by the expression of the α-SMA marker) [124].

### 5.3. Inhibition of the Synthesis of the Farnesyl Group and Farnesylation

As mentioned in the introduction of this review, farnesylation of Ras proteins is necessary for the correct location of Ras in the lipid membranes and the interaction with other molecules such as PI3K. Atorvastatin inhibited the synthesis of the farnesyl groups and its administration reduced the levels of Ras-GTP activation and the induction of kidney fibrosis in a UUO model in mice [124]. The farnesyl-transferase inhibitor L-744,882 reduced Ras-GTP levels and the activation of the ERK1/2 pathway, and these changes resulted in a decrease in ECM synthesis and myofibroblasts abundance in kidneys after the UUO model [124].

Chaetomellic acid is a potent and very specific inhibitor of the farnesyl-transferase, which selectively inhibits H-Ras farnesylation [125]. In the UUO model, the administration of Chaetomellic acid reduced the activation of both ERK1/2 and Akt pathways, but these combined effects were not enough to reduce kidney fibrosis development [124]. In the experimental model of 5/6 renal mass reduction [RMR] in rats, long-term administration of chaetomellic acid decreased the oxidative stress in kidneys [126]. Using the same RMR model, the same authors found a decrease in glomerulosclerosis and arteriolosclerosis after chaetomellic acid administration [127].

### 5.4. MAP Kinase Inhibitors

#### 5.4.1. Inhibition of the ERK1/2 Pathway

As stated previously, the ERK1/2 pathway, downstream of Ras activation, is involved in numerous processes such as tubular cells and myofibroblasts proliferation. Some approaches targeting ERK1/2 have resulted in the amelioration of renal damage in experimental models of renal fibrosis. The inhibition of ERK1/2 with U0126 reduces the renal damage inhibiting mesangial cell proliferation in a rat model of glomerulonephritis [69]. Moreover, ERK1/2 inhibition with PD184352 ameliorated the progression of polycystic kidney disease [128]. While studying tubule-interstitial fibrosis after the UUO model, it was observed that the inhibition of ERK1/2 with U0126 led to a lower myofibroblast abundance, due to a reduction in myofibroblast proliferation evaluated by the presence of their markers α-SMA and vimentin. Moreover, an increase in the proliferation marker Ki67 was observed in tubular cells, suggesting that ERK inhibition induced increases in resistance to atrophy and in tubular preservation after the UUO model [38].

Recently, it has been demonstrated that the MEK inhibitor Trametinib decreases tubulointerstitial fibrosis after the UUO model. Trametinib-induced effects are associated with a decrease in ERK1/2 and AKT activation which reduced myofibroblast expansion after the obstruction [129].

#### 5.4.2. Inhibition of the p38 Pathway

Numerous evidence has related the activity of p38 kinase with ECM deposition in several organs [130], and also with inflammation; p38 MAPK activity is associated with the histological degree of interstitial fibrosis in patients with IgA nephropathy patients. Thus, the inhibition of p38 activity is an interesting therapeutic target for renal fibrosis treatment, as several preclinical studies have suggested.

Moreover, inhibition of p38 with FR167653 resulted in a reduction of kidney fibrosis in a mouse model of CKD with the genetic disorder Nephronophthisis [131].

All these therapeutic strategies summarized in this review have shown their usefulness as tools for the treatment of kidney fibrosis. Nevertheless, numerous preclinical studies will be necessary to continue testing their efficacy, adverse side effects and strategies for drug delivery. On the other hand, these studies provide a good opportunity to consider the expression and activation levels of Ras proteins and their effectors as biomarkers of kidney fibrosis. Regarding the use of Ras proteins and their downstream effectors as potential biomarkers, more studies will be necessary to find correlations between the expression levels of these proteins and the progression of the disease and recovery.

## 6. Conclusions

Several intracellular mediators participate in the regulation of renal fibrosis, TGF-β1 being the cytokine that probably has the greatest relevance, being involved in a high number of pathophysiological circumstances that lead to the accumulation of ECM, both in patients with CKD and in different preclinical experimental models. However, numerous studies in recent years have revealed the important regulatory role of Ras GTPases in the development of different processes that together are responsible for renal fibrosis. This fact highlights the wide range of cellular processes in which these GTPases participate, even though they have been mainly studied in the context of cellular signaling in tumor processes. This review integrates all the available studies to date that show the relevant role of Ras and its effectors in the regulation of pathological kidney fibrosis, and offers a new therapeutic target to act on the evolution or even the possible reversion of renal fibrosis and CKD. This knowledge opens the way to further studies that may develop pharmacological or therapeutic strategies to act on this currently incurable disease.

## Data Availability

Not applicable.
